# New Application of Chloroform in Neuralgia and in Certain Rheumatic Complaints

**Published:** 1861-01

**Authors:** Alex. Sclanders


From the Boston Medical and Surgical Journal.

New Application of Chloroform in Neuralgia and in Certain Rheumatic Complaints.—[At a meeting of the Medico-Chirurgical Society of Edinburgh, Mr. Little, R. F. C. S. E., of Singapore made the following communication, which we reprint from the Edinburgh Medical Journal, for April, 1860.—Eds.]

During my residence at Singapore, East Indies, I was at one time in the habit of using liquor ammonia to produce an immediate blister when instantaneous counter-irritation was thought necessary in certain cerebral affections, &c.—a piece of lint soaked in ammonia being applied to the part, and covered with oil-silk, when in a few minutes so much irritation was produced as to raise a blister. In administering chloroform to my patients, I noticed that their lips were often partially blistered by it; and recollecting the mode of using the ammonia, I thought of trying the chloroform in the same way, but found that neither oil-silk nor gutta percha tissue would answer. I then used a watched-glass to cover the lint soaked in it, and with the best effect.

The manner of application is to take a piece of lint, a little less in size than the watch-glass to be used, (which need not be more than two inches in diameter,) to put it on the hollow side of the glass, to pour on it a few drops of chloroform sufficient to saturate it, and then to apply at once to the part affected, keeping the edges of the glass closely applied to the skin by covering it with the hand, for the purpose of keeping it in position, as well as of assisting the evaporation of the chloroform. This may be done from five to ten minutes, according to the amount of irritation wished for.

The patient during this time will complain of the gradual increase of a burning sensation (not so severe as that produced by a mustard sinapism,) which reaches its height in five minutes, and then abates, but does not entirely disappear for more than ten minutes.

To insure the full operation of the remedy, it is necessary that the watch-glass be rather concave, that it be closely applied to the skin, and that the hand applied over it be sensibly warm. The immediate effect of the application is to remove all local pain in neuralgia, and relieve that of rheumatism.

Its effects on the skin are at first a reddening of the cutis, which in some cases is followed by desquamation of the cuticle; but this depends on the part to which it is applied, and also upon the susceptibility of the individual. In some cases, if the application is prolonged, a dark brown stain remains even for a week or ten days, the same effect as sometimes follows the use of a mustard sinapism.

In Singapore I have used chloroform after this fashion in various neuralgias of the face, in inflammations of the eye and ear, in one case of angina pectoris, in several cases of neuralgia affecting the abdominal parietes, in lumbago, dysmonorrhœa, and in pain attending congestion of the ovary, &c.

Personally I can tëstify to its great efficacy in two severe attacks of rheumatic inflammation of the eyes, in which the pain came on periodically about 3, A. M., with such severity that I thought the loss of sight itself would be preferable to its continuance. All other remedies, such as blisters, leeches, opium externally and internally, belladonna, &c., were of no avail in soothing the pain; water, almost boiling, applied by a sponge, giving only a little relief. I then thought of this use of chloroform, remembering how much it had benefited my patients in other similar affections. The first night the application of it to the temple relieved the pain in ten minutes; and on its return the next night, the application again relieved it; and four times only was it required to remove completely the local pain; allowing, in the meantime, constitutional remedies to produce their effect. Since my return to this country, I have recommended this remedy on several occasions to persons suffering from neuralgia of the face and head, and always with the same good effect as in India; and the other evening one of my domestics was quickly and effectually relieved by it of a painful spasmodic contraction of the platysma myoides muscle, which prevented her raising her head from the chest. The chloroform was applied as directed, with immediate benefit, and next morning she was quite well, though in previous attacks several days elapsed before relieve was obtained. I have mentioned this method to several medical men of this city, who have found it of great benefit; and that it may be more extensively known, is my reason for now bringing it before the profession.

Dr. Keiller mentioned that this plan had been tried with success in his wards.

Dr. Wright has used chloroform for similar purposes, by pouring it into a bottle containing blottling-paper, and applying it over the affected painful part. He has found it sometimes produce vesication, and leave a mark on the skin; but it had been effectual in removing pain.

[Mr. Little has received the following letter from Dr. Sclanders, House Physician to Dr. Keiller in the Royal Infirmary:

ROYAL INFIRMARY, March 14, 1860.

MY DEAR SIR—I have much pleasure in giving you the result of my experience in regard to the external application of chloroform, in the way proposed by you. Soon after you made me aware of it, I saw a friend of mine, who suffered frequently from neuralgia of the left forehead. I proposed the remedy to him, and with effect of immediately removing the pain. Owing to my having kept it too long applied vesication ensued. Since then he has had no return.

I have since used it in several cases of neuralgia of the ovary and pleurodynia, as also in two cases of rheumatic pains in the joints, with marked benefit.

I am, yours truly,

Dr. Little.] Alex. Sclanders.

